# Mitochondrial and Oxidative Stress-Mediated Activation of Protein Kinase D1 and Its Importance in Pancreatic Cancer

**DOI:** 10.3389/fonc.2017.00041

**Published:** 2017-03-15

**Authors:** Heike Döppler, Peter Storz

**Affiliations:** ^1^Department of Cancer Biology, Mayo Clinic Comprehensive Cancer Center, Mayo Clinic, Jacksonville, FL, USA

**Keywords:** protein kinase D, oxidative stress, mitochondria, pancreatic cancer, signaling

## Abstract

Due to alterations in their metabolic activity and decreased mitochondrial efficiency, cancer cells often show increased generation of reactive oxygen species (ROS), but at the same time, to avoid cytotoxic signaling and to facilitate tumorigenic signaling, have mechanism in place that keep ROS in check. This requires signaling molecules that convey increases in oxidative stress to signal to the nucleus to upregulate antioxidant genes. Protein kinase D1 (PKD1), the serine/threonine kinase, is one of these ROS sensors. In this mini-review, we highlight the mechanisms of how PKD1 is activated in response to oxidative stress, so far known downstream effectors, as well as the importance of PKD1-initiated signaling for development and progression of pancreatic cancer.

## Introduction

The Warburg effect in cancer cells is the product of two factors, a return of cells to glycolytic metabolism and increased production of mitochondrial reactive oxygen species (ROS), which is due to alterations in oxidative phosphorylation (1). In established tumors, increased levels of oxidative stress are often accompanied by upregulation of antioxidant systems (2, 3). The upregulation of antioxidant systems keeps ROS at levels where they are protumorigenic and promote cell survival and proliferation, but do not induce apoptosis or necrotic cell death. This mini-review focuses on a ROS-sensing signaling pathway that controls tumor cell detoxification, proliferation, and survival through activation of protein kinase D1 (PKD1).

Protein kinase D1 is one of three members of the PKD family of serine/threonine kinases. PKD1 consists of an *N*-terminal regulatory region and a *C*-terminal kinase domain. Main elements in the regulatory region are two cysteine-rich (C1) domains that are important for lipid binding, and a pleckstrin homology (PH) domain, needed for protein–protein and protein–lipid interactions [reviewed in Ref. (4)]. Dependent on upstream signaling and binding partners, PKDs can be located at various cellular compartments and facilitate Golgi transport processes, as well as mitochondrial, cytosolic, and nuclear signaling [reviewed in Ref. (5)]. An increased oxidative stress leads to PKD1 localization to the mitochondria, where it is activated (6). ROS-activated PKD1 has been shown not only to initiate cytosolic signaling pathways (6–8) but also to redistribute to the nucleus (9). The signaling pathway that leads to the activation of PKD1 by oxidative stress seems unique because it involves tyrosine phosphorylation of the molecule at several residues (8, 10, 11), which do not occur when PKD1 is activated by receptor-mediated signaling (7).

## PKD Activation Downstream of ROS

Protein kinase D1 can be activated by an increase in intracellular oxidative stress levels, such as induced by glutathione depletion or ectopic addition of hydrogen peroxide (7, 8, 12). PKD1 activation also occurs in response to an increase in mitochondrial ROS (mROS) caused by inhibitors of the mitochondrial respiratory chain (13). These include rotenone, a mitochondrial complex I inhibitor, and diphenyleneiodonium, an inhibitor of the NADPH cytochrome P450 reductase (6). Moreover, PKD1 is activated by oncogenes that increase mROS levels such as mutant versions (G12D, G12V) of V-Ki-ras2 Kirsten rat sarcoma viral oncogene homolog (KRas) (14).

Increases in mitochondrial (and cellular) ROS levels initiate a series of tyrosine phosphorylations (Y95, Y432, Y463, and Y502) in PKD1 (8, 10, 11), which are mediated either directly by the proto oncogene tyrosine protein kinase Src or downstream of Src (10, 11). The mechanism of how Src is activated downstream of ROS is not fully understood, and conformational changes due to direct oxidation of cysteine residues, tyrosine nitration, or redox inactivation of inhibitory protein tyrosine phosphatases could be a cause of its increased activity. In this context, it was shown that ROS-responsive receptor-like PTP alpha is required for the activation of PKD1 in response to hydrogen peroxide (15), but a detailed mechanism was not provided. For Src-mediated phosphorylations of PKD1 at Y432 and Y502, no functional consequences have been attributed, so far. Phosphorylation of PKD1 at Y95 is directly mediated by Src (10), whereas Y463 has been shown to be directly phosphorylated by Abelson murine leukemia viral oncogene homolog 1 (Abl), when activated through Src (11).

**Figure 1:**
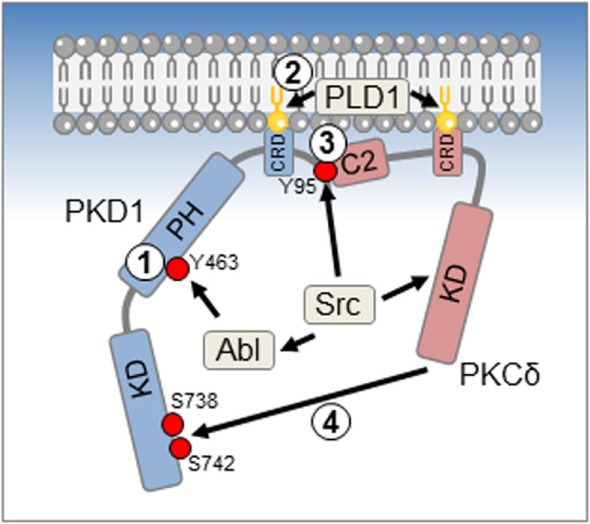
**Reactive oxygen species (ROS)-induced activation mechanism for protein kinase D1 (PKD1)**. An initial event in activation of PKD1 in response to oxidative stress is the phosphorylation at Y463 by Abl (1). This leads to a conformational change in PKD1 that allows docking to membranes such as the outer mitochondrial membrane *via* binding to diacylglycerol (DAG) (2). For mitochondrial membrane anchoring, DAG is generated by ROS-activated phospholipase D1 (PLD1). A third activation step is the phosphorylation of PKD1 at Y95, which is mediated directly by Src (3). This leads to docking of PKCδ *via* its C2 domain and phosphorylation of the PKD1 activation loop serines S738 and S742, rendering PKD1 fully active (4).

A sequential model for activation of PKD1 by ROS has been proposed (Figure 1). The phosphorylation of PKD1 at Y463 in PH domain seems to be an initiating step that leads to a conformational change, which initiates membrane anchoring at the mitochondria (16). This is mediated by binding to diacylglycerol that can be generated through activation of phospholipase D1 downstream of mROS (16). It should be noted that it was also shown that the multifunctional chaperone p32 can act as an adapter that associates PKD1 and PKCδ with mitochondrial membranes (17), but a role for p32 in ROS-initiated activation of PKD1 so far has not been investigated. A next step is the phosphorylation of PKD1 at Y95 by Src. This generates a binding motif for the C2 domain of PKCδ (10), another kinase that is activated downstream of oxidative stress and Src (18). PKCδ then phosphorylates the PKD1 activation loop serines (S738 and S742), resulting in a fully active kinase (7, 10).

## Signaling Through ROS-Activated PKD1 and Functional Consequences

**Figure 2:**
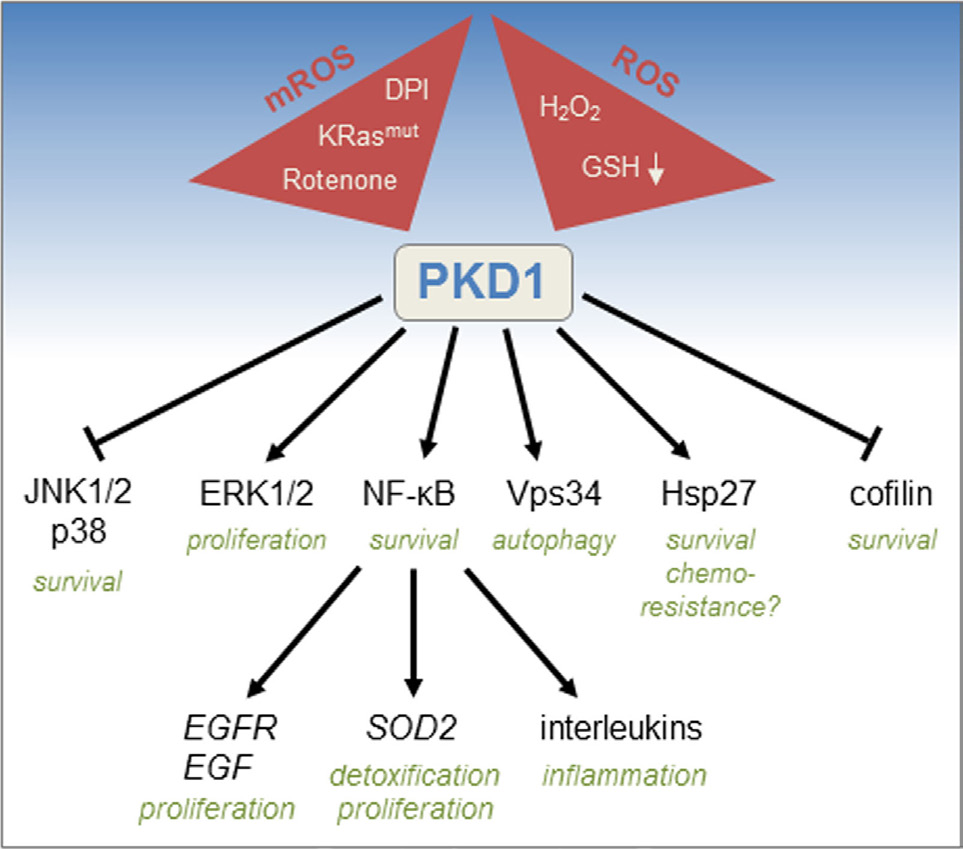
**Mitochondrial reactive oxygen species (mROS)/reactive oxygen species (ROS)-induced activation of protein kinase D1 (PKD1) and downstream signaling**. Activation of PKD1 is mediated by increases in ROS as obtained after ectopically administered hydrogen peroxide (H_2_O_2_) or decrease of glutathione (GSH) or by increases in mROS as obtained by the expression of oncogenic KRas (KRas^mut^), or inhibitors of the mitochondrial respiratory chain such as rotenone and diphenyleneiodonium. ROS-activated PKD1 promotes cell survival by inactivating c-Jun *N*-terminal kinase (JNK) 1/2 and p38 signaling, cofilin function, but also through phosphorylation of Hsp27 and activation of nuclear factor kappa-light-chain-enhancer of activated B cells (NF-κB). PKD1 also promotes proliferation by upregulating extracellular signal-regulated kinases 1/2 (ERK1/2) and epidermal growth factor receptor (EGFR) signaling. Other functions for ROS-activated PKD1 are upregulation of inflammatory cytokines, regulation of autophagy, and chemoresistance.

Several signaling molecules that regulate cell survival and detoxification have been implicated downstream of oxidative stress-activated PKD1 (Figure 2). A main target is the transcription factor nuclear factor kappa-light-chain-enhancer of activated B cells (NF-κB). After activation through the ROS/Src/Abl/PKCδ pathway, PKD1 induces canonical NF-κB signaling through IκB kinase β and subsequent downregulation of inhibitor of kappa-light-chain-enhancer of activated B cells alpha (8). However, the exact molecular mechanisms of how this is facilitated are not known. NF-κB is a protein complex that controls inflammatory signaling, cytokine production, and cell survival. Downstream of PKD1, activation of NF-κB was linked to increased expression of *SOD2*, a gene encoding manganese superoxide dismutase (MnSOD) (6). MnSOD generates hydrogen peroxide, a *bona fide* signaling molecule that is important for tumor cell proliferation (2). PKD1-mediated activation of NF-κB also increases expression of epidermal growth factor receptor (EGFR) and its ligands transforming growth factor alpha (TGFα) and epidermal growth factor (EGF) (14). Besides activation of NF-κB, PKD1 is also involved in other signaling pathways to promote cell survival. For example, in response to oxidative stress, cofilin2 translocates to the mitochondria to interact with the proapoptotic molecule Bax (19). PKD1 inhibits the cofilin phosphatase Slingshot 1L (20), and such signaling attenuates cofilin2 translocation to mitochondria, preserves mitochondrial integrity after oxidative stress, and mediates cell survival (19). Another pathway of how PKD1 promotes cell survival is by activating extracellular signal-regulated kinases 1/2, which confers a protective response to chronic oxidative stress, and by downregulating c-Jun *N*-terminal kinase (JNK) signaling that promotes apoptosis (21, 22). Similarly, downregulation of p38 MAPK signaling by PKD1 in response to hydrogen peroxide has been demonstrated to protect cells from apoptosis (23). Another target for ROS-activated PKD1 is the small heat shock protein Hsp27, which is phosphorylated by PKD1 at S82 (24). PKD1-phosphorylated Hsp27 can bind apoptosis signal-regulating kinase 1 to prevent JNK-induced apoptosis (25). Hsp27 also has been implicated in chemoresistance of several cancers (26, 27). In addition, the tumor suppressor death-associated protein kinase phosphorylates and activates PKD1 in response to oxidative damage (28). Such signaling induces autophagy, due to PKD1-mediated phosphorylation of Vps34, which increases its lipid kinase activity and autophagosome formation (29).

## ROS–PKD1 Signaling in Cancer

Reactive oxygen species–PKD1 signaling has emerged to be important in the pathophysiology of neurodegenerative diseases (30, 31), cardioprotection against ischemia/reperfusion injury (19), tissue inflammation (32), and several cancers, including basal cell carcinoma (33) and pancreatic cancer (14, 34). We here focus on the role of above pathway in pancreatic cancer.

Almost all pancreatic ductal adenocarcinomas (PDAs) are initiated by acquisition of activating *KRAS* mutations (35). During development and progression of PDA, oncogenic KRas protein causes metabolic changes that increase levels of ROS (14, 36–40). KRas-induced suppression of respiratory chain complexes I and III can cause mitochondrial dysfunction and increased generation of mROS (14, 40, 41). Other sources for increased mROS in PDA are enhanced growth factor signaling (42). Oncogenic KRas also activate nuclear respiratory factor 2 to upregulate antioxidant systems to counterbalance the increases in oxidative stress (14, 43). This is accompanied by an upregulation of the *SOD2* gene, whose gene product, MnSOD, leads to formation of hydrogen peroxide (44). In sum, the upregulation of antioxidant enzymes keeps ROS at levels where they are protumorigenic (3, 14, 45, 46). Further depletion of KRas-caused mROS decreases pancreatic tumorigenesis in genetic animal models (14, 45).

Although in normal fibroblast cells, the ROS/PKCδ/PKD1 pathway downstream of oncogenic KRas upregulates pro-inflammatory signaling (expression of interleukin-6 and interleukin-8) and may contribute to senescence (47), under pathophysiological conditions, this pathway drives initiation of PDA. For example, after pancreatic inflammation (pancreatitis), PKCδ/PKD1/NF-κB signaling is induced in pancreatic acinar cells (48) and contributes to acinar-to-ductal metaplasia, a process that leads to pancreatic lesions (34). In the presence of an oncogenic KRas mutation, these lesions can then further develop to pancreatic cancer. KRas/mROS/PKD1/NF-κB signaling contributes to tumor initiation by upregulating expression of EGFR and its ligands TGFα and EGF (14). EGFR signaling then elevates overall (oncogenic and wild-type) KRas activity to pathological levels (49–51). Another role for PKD1 during initiation of pancreatic cancer is the activation of Notch signaling downstream of mutant KRas (34). Although there is no direct evidence that PKD1/Notch signaling is due to production of mROS, Notch and NF-κB pathways have been shown to co-operate in processes that mediate development of PDA (52).

## Conclusion

The occurrence of increased oxidative stress in tumor cells requires ROS-sensing signaling to upregulate antioxidant systems to counterbalance ROS. This opens an opportunity for targeting tumor cells (46). In response to ROS, PKD1 has been shown to regulate prosurvival and proliferation signaling through various factors (Figure 2). In addition, PKD1 signaling also determines the threshold of mitochondrial depolarization that leads to the production of ROS (53). Therefore, targeting PKD1 or PKD1 downstream signaling may be efficient to drive ROS to levels where they are toxic for cancer cells. In recent years, a variety of PKD inhibitors have been developed and successfully tested in preclinical models. For example, for othotopically implanted pancreatic cancer cells, the PKD inhibitor CRT0066101 showed promising effects on primary tumors (54). However, it is not known if this inhibitor can be used for late stage tumors, or if it will show efficacy in combination therapy with currently used chemotherapeutics. Clearly, additional studies are needed to fully evaluate the value of targeting ROS-PKD signaling for cancer therapy.

## Author Contributions

Both authors have made equal intellectual contributions to text and figures.

## Conflict of Interest Statement

The authors declare that the research was conducted in the absence of any commercial or financial relationships that could be construed as a potential conflict of interest.

## References

[1] Wallace DC. Mitochondria and cancer: Warburg addressed. Cold Spring Harb Symp Quant Biol (2005) 70:363–74. 10.1101/sqb.2005.70.035 16869773

[2] Liou GY Storz P Reactive oxygen species in cancer. Free Radic Res (2010) 44(5):479–96. 10.3109/10715761003667554 20370557 PMC3880197

[3] Storz P. KRas, ROS and the initiation of pancreatic cancer. Small GTPases (2017) 8(1):38–42. 10.1080/21541248.2016.1192714 27215184 PMC5331894

[4] Rozengurt E Protein kinase D signaling: multiple biological functions in health and disease. Physiology (Bethesda) (2011) 26(1):23–33. 10.1152/physiol.00037.2010 21357900 PMC4381749

[5] Fu Y Rubin CS. Protein kinase D: coupling extracellular stimuli to the regulation of cell physiology. EMBO Rep (2011) 12(8):785–96. 10.1038/embor.2011.139 21738220 PMC3147268

[6] Storz P Doppler H Toker A. Protein kinase D mediates mitochondrion-to-nucleus signaling and detoxification from mitochondrial reactive oxygen species. Mol Cell Biol (2005) 25(19):8520–30. 10.1128/MCB.25.19.8520-8530.2005 16166634 PMC1265746

[7] Storz P Doppler H Toker A. Protein kinase Cdelta selectively regulates protein kinase D-dependent activation of NF-kappaB in oxidative stress signaling. Mol Cell Biol (2004) 24(7):2614–26. 10.1128/MCB.24.7.2614-2626.2004 15024053 PMC371115

[8] Storz P Toker A. Protein kinase D mediates a stress-induced NF-kappaB activation and survival pathway. EMBO J (2003) 22(1):109–20. 10.1093/emboj/cdg009 12505989 PMC140053

[9] Waldron RT Rey O Zhukova E Rozengurt E. Oxidative stress induces protein kinase C-mediated activation loop phosphorylation and nuclear redistribution of protein kinase D. J Biol Chem (2004) 279(26):27482–93. 10.1074/jbc.M402875200 15084589

[10] Doppler H Storz P. A novel tyrosine phosphorylation site in protein kinase D contributes to oxidative stress-mediated activation. J Biol Chem (2007) 282(44):31873–81. 10.1074/jbc.M703584200 17804414

[11] Storz P Doppler H Johannes FJ Toker A. Tyrosine phosphorylation of protein kinase D in the pleckstrin homology domain leads to activation. J Biol Chem (2003) 278(20):17969–76. 10.1074/jbc.M213224200 12637538

[12] Waldron RT Rozengurt E. Oxidative stress induces protein kinase D activation in intact cells. Involvement of Src and dependence on protein kinase C. J Biol Chem (2000) 275(22):17114–21. 10.1074/jbc.M908959199 10748111

[13] Li N Ragheb K Lawler G Sturgis J Rajwa B Melendez JA Mitochondrial complex I inhibitor rotenone induces apoptosis through enhancing mitochondrial reactive oxygen species production. J Biol Chem (2003) 278(10):8516–25. 10.1074/jbc.M210432200 12496265

[14] Liou GY Doppler H DelGiorno KE Zhang L Leitges M Crawford HC Mutant KRas-induced mitochondrial oxidative stress in acinar cells upregulates EGFR signaling to drive formation of pancreatic precancerous lesions. Cell Rep (2016) 14(10):2325–36. 10.1016/j.celrep.2016.02.029 26947075 PMC4794374

[15] Hao Q Rutherford SA Low B Tang H. Selective regulation of hydrogen peroxide signaling by receptor tyrosine phosphatase-alpha. Free Radic Biol Med (2006) 41(2):302–10. 10.1016/j.freeradbiomed.2006.04.011 16814111

[16] Cowell CF Doppler H Yan IK Hausser A Umezawa Y Storz P. Mitochondrial diacylglycerol initiates protein-kinase D1-mediated ROS signaling. J Cell Sci (2009) 122(Pt 7):919–28. 10.1242/jcs.041061 19258390 PMC2720927

[17] Storz P Hausser A Link G Dedio J Ghebrehiwet B Pfizenmaier K Protein kinase C [micro] is regulated by the multifunctional chaperon protein p32. J Biol Chem (2000) 275(32):24601–7. 10.1074/jbc.M002964200 10831594

[18] Steinberg SF Mechanisms for redox-regulation of protein kinase C. Front Pharmacol (2015) 6:128 10.3389/fphar.2015.00128 26157389 PMC4477140

[19] Xiang SY Ouyang K Yung BS Miyamoto S Smrcka AV Chen J PLCepsilon, PKD1, and SSH1L transduce RhoA signaling to protect mitochondria from oxidative stress in the heart. Sci Signal (2013) 6(306):ra108 10.1126/scisignal.2004405 24345679 PMC4035240

[20] Eiseler T Doppler H Yan IK Kitatani K Mizuno K Storz P. Protein kinase D1 regulates cofilin-mediated F-actin reorganization and cell motility through slingshot. Nat Cell Biol (2009) 11(5):545–56. 10.1038/ncb1861 19329994 PMC2761768

[21] Singh R Czaja MJ Regulation of hepatocyte apoptosis by oxidative stress. J Gastroenterol Hepatol (2007) 22(Suppl 1):S45–8. 10.1111/j.1440-1746.2006.04646.x 17567464

[22] Wang Y Schattenberg JM Rigoli RM Storz P Czaja MJ. Hepatocyte resistance to oxidative stress is dependent on protein kinase C-mediated down-regulation of c-Jun/AP-1. J Biol Chem (2004) 279(30):31089–97. 10.1074/jbc.M404170200 15145937

[23] Song J Li J Qiao J Jain S Mark Evers B Chung DH. PKD prevents H2O2-induced apoptosis via NF-kappaB and p38 MAPK in RIE-1 cells. Biochem Biophys Res Commun (2009) 378(3):610–4. 10.1016/j.bbrc.2008.11.106 19059215 PMC2631172

[24] Doppler H Storz P Li J Comb MJ Toker A. A phosphorylation state-specific antibody recognizes Hsp27, a novel substrate of protein kinase D. J Biol Chem (2005) 280(15):15013–9. 10.1074/jbc.C400575200 15728188

[25] Sharp FR Zhan X Liu DZ. Heat shock proteins in the brain: role of Hsp70, Hsp 27, and HO-1 (Hsp32) and their therapeutic potential. Transl Stroke Res (2013) 4(6):685–92. 10.1007/s12975-013-0271-4 24323422 PMC3858824

[26] Liu QH Zhao CY Zhang J Chen Y Gao L Ni CY Role of heat shock protein 27 in gemcitabine-resistant human pancreatic cancer: comparative proteomic analyses. Mol Med Rep (2012) 6(4):767–73. 10.3892/mmr.2012.1013 22858734

[27] Lu H Sun C Zhou T Zhou B Guo E Shan W HSP27 knockdown increases cytoplasmic p21 and cisplatin sensitivity in ovarian carcinoma cells. Oncol Res (2016) 23(3):119–28. 10.3727/096504015X14496932933656 26931434 PMC7838724

[28] Eisenberg-Lerner A Kimchi A. DAP kinase regulates JNK signaling by binding and activating protein kinase D under oxidative stress. Cell Death Differ (2007) 14(11):1908–15. 10.1038/sj.cdd.4402212 17703233

[29] Eisenberg-Lerner A Kimchi A. PKD is a kinase of Vps34 that mediates ROS-induced autophagy downstream of DAPk. Cell Death Differ (2012) 19(5):788–97. 10.1038/cdd.2011.149 22095288 PMC3321617

[30] Asaithambi A Ay M Jin H Gosh A Anantharam V Kanthasamy A Protein kinase D1 (PKD1) phosphorylation promotes dopaminergic neuronal survival during 6-OHDA-induced oxidative stress. PLoS One (2014) 9(5):e96947. 10.1371/journal.pone.0096947 24806360 PMC4013052

[31] Ay M Jin H Harischandra DS Asaithambi A Kanthasamy A Anantharam V Molecular cloning, epigenetic regulation, and functional characterization of Prkd1 gene promoter in dopaminergic cell culture models of Parkinson’s disease. J Neurochem (2015) 135(2):402–15. 10.1111/jnc.13261 26230914 PMC4839478

[32] Thrower EC Yuan J Usmani A Liu Y Jones C Minervini SN A novel protein kinase D inhibitor attenuates early events of experimental pancreatitis in isolated rat acini. Am J Physiol Gastrointest Liver Physiol (2011) 300(1):G120–9. 10.1152/ajpgi.00300.2010 20947701 PMC3025506

[33] Arun SN Kaddour-Djebbar I Shapiro BA Bollag WB. Ultraviolet B irradiation and activation of protein kinase D in primary mouse epidermal keratinocytes. Oncogene (2011) 30(13):1586–96. 10.1038/onc.2010.540 21132013 PMC3069139

[34] Liou GY Doppler H Braun UB Panayiotou R Scotti Buzhardt M Radisky DC Protein kinase D1 drives pancreatic acinar cell reprogramming and progression to intraepithelial neoplasia. Nat Commun (2015) 6:6200. 10.1038/ncomms7200 25698580 PMC4394184

[35] Hruban RH Iacobuzio-Donahue C Wilentz RE Goggins M Kern SE Molecular pathology of pancreatic cancer. Cancer J (2001) 7(4):251–8. 11561601

[36] Ahn CS Metallo CM. Mitochondria as biosynthetic factories for cancer proliferation. Cancer Metab (2015) 3(1):1. 10.1186/s40170-015-0128-2 25621173 PMC4305394

[37] Kodydkova J Vavrova L Stankova B Macasek J Krechler T Zak A. Antioxidant status and oxidative stress markers in pancreatic cancer and chronic pancreatitis. Pancreas (2013) 42(4):614–21. 10.1097/MPA.0b013e318288360a 23558240

[38] Son J Lyssiotis CA Ying H Wang X Hua S Ligorio M Glutamine supports pancreatic cancer growth through a KRAS-regulated metabolic pathway. Nature (2013) 496(7443):101–5. 10.1038/nature12040 23535601 PMC3656466

[39] Vander Heiden MG Cantley LC Thompson CB Understanding the Warburg effect: the metabolic requirements of cell proliferation. Science (2009) 324(5930):1029–33. 10.1126/science.1160809 19460998 PMC2849637

[40] Weinberg F Hamanaka R Wheaton WW Weinberg S Joseph J Lopez M Mitochondrial metabolism and ROS generation are essential for Kras-mediated tumorigenicity. Proc Natl Acad Sci U S A (2010) 107(19):8788–93. 10.1073/pnas.1003428107 20421486 PMC2889315

[41] Hu Y Lu W Chen G Wang P Chen Z Zhou Y K-ras(G12V) transformation leads to mitochondrial dysfunction and a metabolic switch from oxidative phosphorylation to glycolysis. Cell Res (2012) 22(2):399–412. 10.1038/cr.2011.145 21876558 PMC3257361

[42] Korc M Role of growth factors in pancreatic cancer. Surg Oncol Clin N Am (1998) 7(1):25–41. 9443985

[43] DeNicola GM Karreth FA Humpton TJ Gopinathan A Wei C Frese K Oncogene-induced Nrf2 transcription promotes ROS detoxification and tumorigenesis. Nature (2011) 475(7354):106–9. 10.1038/nature10189 21734707 PMC3404470

[44] Song JH An N Chatterjee S Kistner-Griffin E Mahajan S Mehrotra S Deletion of Pim kinases elevates the cellular levels of reactive oxygen species and sensitizes to K-Ras-induced cell killing. Oncogene (2015) 34(28):3728–36. 10.1038/onc.2014.306 25241892 PMC4369476

[45] Al Saati T Clerc P Hanoun N Peuget S Lulka H Gigoux V Oxidative stress induced by inactivation of TP53INP1 cooperates with KrasG12D to initiate and promote pancreatic carcinogenesis in the murine pancreas. Am J Pathol (2013) 182(6):1996–2004. 10.1016/j.ajpath.2013.02.034 23578383

[46] Durand N Storz P Targeting reactive oxygen species in development and progression of pancreatic cancer. Expert Rev Anticancer Ther (2017) 17(1):19–31. 10.1080/14737140.2017.1261017 27841037 PMC5518736

[47] Wang P Han L Shen H Wang P Lv C Zhao G Protein kinase D1 is essential for Ras-induced senescence and tumor suppression by regulating senescence-associated inflammation. Proc Natl Acad Sci U S A (2014) 111(21):7683–8. 10.1073/pnas.1310972111 24828530 PMC4040603

[48] Yuan J Lugea A Zheng L Gukovsky I Edderkaoui M Rozengurt E Protein kinase D1 mediates NF-kappaB activation induced by cholecystokinin and cholinergic signaling in pancreatic acinar cells. Am J Physiol Gastrointest Liver Physiol (2008) 295(6):G1190–201. 10.1152/ajpgi.90452.2008 18845574 PMC2604803

[49] Ardito CM Gruner BM Takeuchi KK Lubeseder-Martellato C Teichmann N Mazur PK EGF receptor is required for KRAS-induced pancreatic tumorigenesis. Cancer Cell (2012) 22(3):304–17. 10.1016/j.ccr.2012.07.024 22975374 PMC3443395

[50] Huang H Daniluk J Liu Y Chu J Li Z Ji B Oncogenic K-Ras requires activation for enhanced activity. Oncogene (2014) 33(4):532–5. 10.1038/onc.2012.619 23334325 PMC3923400

[51] Navas C Hernandez-Porras I Schuhmacher AJ Sibilia M Guerra C Barbacid M. EGF receptor signaling is essential for k-ras oncogene-driven pancreatic ductal adenocarcinoma. Cancer Cell (2012) 22(3):318–30. 10.1016/j.ccr.2012.08.001 22975375 PMC3601542

[52] Maniati E Bossard M Cook N Candido JB Emami-Shahri N Nedospasov SA Crosstalk between the canonical NF-kappaB and Notch signaling pathways inhibits Ppargamma expression and promotes pancreatic cancer progression in mice. J Clin Invest (2011) 121(12):4685–99. 10.1172/JCI45797 22056382 PMC3225987

[53] Zhang T Sell P Braun U Leitges M PKD1 protein is involved in reactive oxygen species-mediated mitochondrial depolarization in cooperation with protein kinase Cdelta (PKCdelta). J Biol Chem (2015) 290(16):10472–85. 10.1074/jbc.M114.619148 25759386 PMC4400355

[54] Harikumar KB Kunnumakkara AB Ochi N Tong Z Deorukhkar A Sung B A novel small-molecule inhibitor of protein kinase D blocks pancreatic cancer growth in vitro and in vivo. Mol Cancer Ther (2010) 9(5):1136–46. 10.1158/1535-7163.MCT-09-1145 20442301 PMC2905628

